# Synergistic application of melatonin and methyl jasmonate mitigates drought-induced oxidative and photosynthetic impairment in *Brassica napus*

**DOI:** 10.1080/15592324.2026.2630126

**Published:** 2026-02-11

**Authors:** Hamzeh Amiri, Parvaneh Hemmati Hassan Gavyar

**Affiliations:** a Department of Biology, Faculty of Basic Science, Lorestan University, Khorramabad, Iran

**Keywords:** Abiotic stress, reactive oxygen species, phytohormones, stomatal conductance, oxidative damage, *Brassica napus*

## Abstract

Drought stress severely limits rapeseed (Brassica napus L.) growth and productivity by disrupting photosynthetic efficiency and inducing oxidative damage. This study investigated the potential of melatonin (MT) and methyl jasmonate (MJ), individually and in combination, to mitigate drought-induced toxicity in rapeseed. Plants were subjected to drought stress (15% Polyethylene glycol 6000), followed by foliar application of MT (100 μM), MJ (100 μM), or their combination. Under drought stress, rapeseed exhibited significantly reduced chlorophyll content (by 33.72% for chl a and 54.59% for chl b), impaired photosystem II efficiency (Fv/Fm decreased by 16.08%), and elevated oxidative stress markers (H₂O₂ increased by 54.79%, MDA by 144.6%, ROS by 43.96%). The combined application of MT and MJ conferred the highest level of drought tolerance, increasing shoot fresh weight by 92.33% and root dry weight by 405% compared to the stressed control. This synergistic treatment effectively enhanced stomatal conductance by 76.25%, increased net photosynthetic rate (Pn) by 71.45%, and boosted key antioxidant enzyme activities (e.g., POD by 216%). The enhanced efficacy of the dual treatment compared to individual applications strongly indicates coordinated signaling between MT and MJ pathways in stress response modulation. These results demonstrate that simultaneous application of these plant growth regulators offers an effective physiological strategy to improve rapeseed performance under water deficit conditions.

## 1. Introduction

Agriculture is responsible for providing food for a world whose population is increasing every year, while the area of cultivated land globally remains almost constant. Additionally, biotic and abiotic stresses lead to reduced agricultural productivity. Among abiotic stresses, drought is the most significant factor limiting plant growth, as water availability greatly influences plant development. Drought stress causes changes in growth, photosynthetic, and biochemical parameters of plants.^1^ This stress results in reduced transpiration, stomatal closure, decreased relative water content, decreased photosynthetic pigments (chlorophylls and carotenoids), as well as decreases in Pn and Fv/Fm, reduced canopy size, increased osmolytes, and changes in the activity of enzymatic and non-enzymatic antioxidants.^2^^,^^3^ When a plant is under stress, indicators of oxidative damage, including hydrogen peroxide content, malondialdehyde, and ion leakage, increase. In these conditions, reactive oxygen species such as hydrogen peroxide and superoxide anion accumulate in cells, react with cellular membranes, and cause damage to intracellular organelles. Reactive oxygen species affect the chloroplastic and mitochondrial electron transport chains through lipid peroxidation, potentially leading to plant death. However, the impact of drought stress on plants heavily depends on the severity of the stress and the growth stage of the plant.^2^

Rapeseed (Brassica napus L.), a cornerstone of global edible oil and biofuel production, faces significant threats from drought stress, which is increasingly prevalent due to climate change. Water deficit induces a complex array of physiological dysfunctions that ultimately manifest as substantial yield losses. The reproductive stages of rapeseed are exceptionally vulnerable; drought during flowering and pod development drastically increases flower and pod abortion, directly reducing the number of siliques per plant.^4^ Furthermore, impaired seed filling, a consequence of disrupted assimilate partitioning under stress, leads to fewer seeds per pod and reduced individual seed weight, compromising both yield and oil quality.^5^ The physiological cascade begins with stomatal closure, a primary response to conserve water that simultaneously restricts carbon dioxide uptake, thereby inhibiting photosynthesis.^6^ This reduction in photosynthetic capacity limits the supply of carbohydrates essential for growth and seed development. Concurrently, drought disrupts plant-water relations, lowering tissue water potential and relative water content, which curtails cell expansion and growth. In response, rapeseed may activate osmotic adjustment mechanisms, accumulating solutes like proline to maintain turgor, a key trait associated with drought tolerance.^7^ A critical secondary effect of impaired photosynthesis and electron transport is the accumulation of reactive oxygen species (ROS), causing oxidative damage to lipids, proteins, and DNA, which further disrupts cellular homeostasis.^8^ These ROS are often mitigated by the upregulation of antioxidant enzymes. Nutrient uptake and translocation are also hindered under drought due to reduced transpirational flow and root activity, potentially inducing deficiencies that compound plant stress.^9^ Underpinning these physiological changes are pronounced hormonal shifts, most notably a surge in abscisic acid (ABA), which coordinates drought responses including stomatal regulation and the expression of stress-responsive genes.^10^

Given the adverse effects of drought stress on plants, it is essential to provide solutions for enhancing stress resistance and increasing agricultural productivity. Recently, the use of plant growth regulators, including melatonin (MT) and methyl jasmonate (MJ), has attracted researchers' attention to mitigate the adverse environmental effects on plants. In fact, plant growth regulators are organic or inorganic compounds that affect physiological processes in plants at low concentrations.^11^ MT is an amphiphilic indole amine with a low molecular weight, derived from tryptophan, capable of passing through cellular membranes.^12^ MT can act as an antioxidant molecule, protecting cells from oxidative damage and increasing plant resistance to environmental stresses.^13^ Studies have shown that this regulator enhances the resistance of plants to abiotic stresses such as heat, drought, salinity, low temperature, and heavy metals.^14^ Research indicates that during evolution, MT has changed its biological role from an antioxidant to a signaling hormone.^13^ Regardless, this regulator, whether through its antioxidant role or signaling role, has activated plant defense mechanisms and pathways, enhancing stress tolerance by modulating physiological, antioxidant, and biochemical mechanisms in plants.^2^^,^^15^

Jasmonic acid and its derivative methyl jasmonate are among the important phytohormones that respond to environmental stresses.^16^ One of the primary responses of plants to drought stress is stomatal closure. Reports indicate that MJ, under stress conditions, facilitates the exit of ions from guard cells through calcium and anion channels, resulting in stomatal closure.^17^ Most abiotic stresses often lead to oxidative damage. Studies have shown that MJ reduces oxidative damage by activating plant defense systems, including increasing malondialdehyde and antioxidant systems, thus protecting the photosynthetic apparatus and chlorophyll reservoirs of the plant, and enhancing plant resistance to stresses such as drought, salinity, and heavy metals.^16^ Furthermore, the external application of this growth regulator under stress conditions helps maintain the relative water content of the plant and increases stress resistance.^18^

Rapeseed or canola, scientifically known as *Brassica napus* and belonging to the Brassicaceae family, is one of the most important and valuable crops in the world.^19^ Various parts of this plant, including flowers, seeds, leaves, stems, and roots, are used in the food, pharmaceutical, and cosmetic industries. The seeds of rapeseed are the most valuable part of the plant due to their high protein and oil content. Additionally, the seeds contain glucosinolates, phenols, phytic acids, cellulose, and sugars.^20^

Considering the increasing spread of drought globally, especially in Iran, studying the physiological responses of plants to water stress especially in economically and nutritionally valuable crops like rapeseed is of great importance. Moreover, given the constant area of cultivated land against the growing global population, providing innovative methods to enhance the quantity and quality of agricultural products is essential. The aim of this study is to investigate the antioxidant and photosynthetic responses of rapeseed to drought stress and the potential role of MT and MJ as novel growth regulators in increasing rapeseed's tolerance to water stress.

## 2. Materials and methods

### 2.1. Plant material and treatments

A pot experiment was conducted at the research greenhouse of Lorestan University as a factorial experiment in a completely randomized design (CRD). Seeds of canola (RGS cultivar) were sterilized using a 5% sodium hypochlorite solution before being grown in perlite-filled pots. These pots were subsequently transferred to a growth chamber set to maintain 75% relative humidity, a photoperiod of 14 hours light and 10 hours darkness, and day/night temperatures of 25 ± 1 °C and 17 ± 1 °C, respectively. Ten seeds were sown in each pot, which were then thinned to four uniform plants at the seedling stage. The emerging seedlings were irrigated exclusively with distilled water until the leaf emergence stage, after which they were gradually introduced to a half-strength Hoagland's nutrient solution. For the subsequent four weeks until the experiment's conclusion, the plants were consistently maintained on this half-strength solution, which was applied at regular intervals. A drought stress treatment was initiated in the fourth week using a 15% (w/v) solution of polyethylene glycol 6000. Foliar sprays of MT and MJ (100 μM) were administered five times in a single day at two-hour intervals, ensuring complete leaf coverage. The experimental pots were categorized into eight distinct groups with three repetitions: Group 1 served as the control with no stress or hormone application; Group 2 was subjected solely to drought stress; Group 3 received only foliar MT without stress; Group 4 was exposed to drought stress along with foliar MT; Group 5 was treated with foliar MJ only; Group 6 underwent drought stress combined with MJ; Group 7 received a combined foliar application of both MT and MJ without stress; and Group 8 experienced drought stress along with the combined hormone treatment (Table 1). In the twelfth week, two-thirds of the roots and aerial parts of the plant were gathered and immediately frozen in liquid nitrogen and stored at –80 °C for subsequent analysis, and one-third remaining were used to determine the fresh and dry weight of plants.

**Table 1.** Grouping of applied treatments.

Groups	PEG (15%)	MT(100 μM)	MJ(100 μM)
1	−	−	−
2	+	−	−
3	−	+	−
4	+	+	−
5	−	−	+
6	+	−	+
7	−	+	+
8	+	+	+

PEG: Polyethylene glycol; MT: Melatonin, MJ: Methyl jasmonate.

### 2.2. Preparation of MT and MJ

#### 2.2.1. Preparation of MT

The MT solution was formulated according to the method described by Ye et al.^21^, with minor modifications. Briefly, a stock solution was produced by dissolving 0.02328 g of MT in 506.1 μL of pure ethanol. For the working solution, 101.2 μL of this stock was then diluted in 200 mL of distilled water, with 0.05% (v/v) Tween 20 added as a wetting agent.

#### 2.2.2. Preparation of MJ

The MJ solution was formulated according to the method described by Hamidian et al.^22^, with minor modifications. Briefly, a stock solution was produced by dissolving 0.00448 g of MJ in 1000 μL of pure ethanol. Then, the volume was made up to 200 ml with distilled water, with 0.05% (v/v) Tween 20 added as a wetting agent.

### 2.3. Growth parameters and RWC

The fresh weight (FW) of the plant samples was recorded immediately upon harvest. To determine the dry weight (DW), the samples were then placed in an oven at 70 °C for 48 hours until a constant weight was achieved.^23^ For the assessment of Relative Water Content (RWC), the first fresh leaves were saturated by immersion in distilled water for 24 hours to obtain their turgid weight (TW). These same samples were then oven-dried at 70 °C for 48 hours to find their DW. The RWC was subsequently calculated based on the formula provided by Martínez et al.^24^: RWC = [(FW − DW)/(TW − DW)] × 100.

### 2.4. Analysis of photosynthetic properties

Photosynthetic pigment concentrations (chlorophyll a, b, and carotenoids) were extracted from fresh leaf tissue using 80% acetone and quantified spectrophotometrically based on followed equations.^25^

Chlorophyll a (mg/gFW) = [12.7 (OD663)−2.69(OD645)] × V/1000 × wt.

Chlorophyll b (mg/gFW) = [22.9 (OD645)−4.68(OD663)] × V/1000 × wt.

Carotenoids (mg/gFW) = [(1000 × OD470)−(3.27 × mg chl a)−[(104 × mg chl b)]/227.

Where; OD: optical density at certain wave length (645, 470 or 663 nm), v- final volume wt: weight of sample.

During the ninth week of the experiment, measurements of gas exchange and chlorophyll fluorescence were taken. A portable CI-340 photosynthesis system (CID, USA) was used to record gas exchange parameters in the leaves by monitoring CO_2_ uptake. Chlorophyll fluorescence was assessed using a Pocket PEA fluorimeter (UK) at ambient temperature. Prior to measurement, leaves were adapted to darkness for 20 minutes using specialized leaf clips, after which the fluorescence signal was recorded.

### 2.5. Biochemical analyzes

#### 2.5.1. Assessment of compatible solutes

Proline levels were analyzed via a ninhydrin assay.^26^ Briefly, 0.3 g of leaf tissue was ground in 1 mL of 3% sulfosalicylic acid and centrifuged at 12,766 × g for 20 minutes. A portion of the resulting supernatant was combined with ninhydrin and glacial acetic acid, heated at 100 °C for 60 minutes, and then cooled. After adding toluene and vortexing, the absorbance of the toluene layer was read at 520 nm.

The concentration of total soluble carbohydrates (TSCs) was measured based on a published protocol.^27^ Leaf samples (0.01 g) were homogenized in 1 mL of 10 mM phosphate buffer (pH 7.0) and centrifuged. Then, 100 µL of the supernatant was added to 300 µL of concentrated sulfuric acid, vortexed, and cooled on ice. Absorbance was taken at 315 nm, and concentrations were derived from a glucose standard curve.

Total reducing sugars were quantified with the dinitrosalicylic acid (DNS) method.^28^ The DNS reagent was made by dissolving 1 g of DNS and 30 g of sodium potassium tartrate in 80 mL of 0.5 M KOH with mild heating, then bringing the volume to 100 mL with water. For the test, 0.01 g of leaf material was homogenized in phosphate buffer and centrifuged. A 100 µL aliquot of the supernatant was mixed with 400 µL of DNS reagent, heated to 95 °C for 5 minutes, cooled to 25 °C, and absorbance was measured at 540 nm.

#### 2.5.2. Assays for oxidative stress markers and membrane stability

The generation rate of reactive oxygen species (ROS) was evaluated using dichlorofluorescein diacetate (DCFH-DA) following Gavyar et al.^29^ The assay mixture (1.82 mL total) contained 1.7 mL of 50 mM phosphate buffer (pH 7.4), 20 µL of plant extract, and 100 µL of 10 µM DCFH-DA. After incubation at 37 °C for 15 minutes, fluorescence was measured with excitation at 488 nm and emission at 521 nm.

Hydrogen peroxide (H₂O₂) was quantified according to Alexieva et al.^30^ Leaf material (0.3 g) was homogenized in 1 mL of 0.1% trichloroacetic acid (TCA) and centrifuged. A 200 µL sample of the supernatant was reacted with 50 µL of 10 mM phosphate buffer and 200 µL of 1 M potassium iodide. Following a 1-hour dark incubation, absorbance was recorded at 390 nm, and concentration was calculated using an extinction coefficient of 0.28 mM^−1^ cm^−1^.

The extent of lipid peroxidation was estimated as malondialdehyde (MDA) content via the thiobarbituric acid (TBA) reaction.^31^ Fresh leaf (0.5 g) was homogenized in 1 mL of 0.1% TCA and centrifuged. A 250 µL portion of the supernatant was combined with 500 µL of 0.5% TBA (in 20% TCA), heated at 95 °C for 30 minutes, quickly cooled on ice, and centrifuged again. Absorbance readings were taken at 532 nm and 600 nm. MDA concentration was determined using the difference in absorbance (A₅₃₂−A₆₀₀) and an extinction coefficient of 155 mM^−1^ cm^−1^.

Membrane integrity was evaluated by measuring electrolyte leakage (EL) as described by Lutts et al.^32^ Leaf segments (0.2 g) were washed and placed in 10 mL of deionized water for 24 hours at room temperature. The initial electrical conductivity (EC₁) of the solution was measured. Samples were then autoclaved at 120 °C for 20 minutes, cooled, and the final conductivity (EC₂) was recorded. EL was calculated as a percentage: (EC₁/EC₂) × 100.

#### 2.5.3. Preparation of enzyme extract

To assay antioxidant enzymes, a crude extract was obtained by homogenizing 0.3 g of fresh leaf tissue with 1.5 mL of a chilled extraction buffer. The buffer was composed of 2-mercaptoethanol, glycerol, phosphate buffer, and EDTA in a 1:1:3:1 volumetric ratio. The homogenate was centrifuged at 12,766 × g for 20 minutes at 4 °C, and the collected supernatant served as the enzyme source.

#### 2.5.4. Evaluation of antioxidant systems

##### 2.5.4.1. Enzymatic antioxidants.

Catalase (CAT) activity was determined by tracking the disappearance of H₂O₂ at 240 nm over one minute.^33^ The 1.5 mL reaction volume included 50 µL of enzyme extract and 1.45 mL of assay buffer (50 mM phosphate buffer, pH 7.0, with 70 mM H₂O₂).

Ascorbate peroxidase (APX) activity was assayed by monitoring ascorbate oxidation at 290 nm for one minute.^34^ The 1 mL reaction mixture contained 100 µL of enzyme extract and 900 µL of assay buffer with ascorbate and H₂O₂.

Guaiacol peroxidase (POD) activity was measured.^35^ The 1 mL reaction consisted of 475 µL of 45 mM guaiacol, 475 µL of 100 mM H₂O₂, and 50 µL of enzyme extract. The increase in absorbance at 470 nm from guaiacol oxidation was recorded for 2 minutes.

Superoxide dismutase (SOD) activity was assessed by its inhibition of nitroblue tetrazolium (NBT) photoreduction at 560 nm.^36^ The 1 mL reaction mixture contained 100 µL of enzyme extract and 900 µL of a solution with methionine, NBT, and riboflavin.

##### 2.5.4.2. Non-enzymatic antioxidants.

For non-enzymatic assays, an ethanolic extract was prepared by homogenizing 0.15 g of leaf tissue in 1 mL of 95% ethanol, followed by centrifugation at 12,766 × g for 20 minutes.

Total phenolic content was analyzed with the Folin-Ciocalteu reagent.^37^ A supernatant aliquot was mixed with diluted Folin-Ciocalteu reagent and sodium carbonate solution. After 2 hours in the dark, absorbance was measured at 760 nm. Concentration was calculated using a gallic acid standard curve (Absorbance = 0.0016 × Gallic acid (µg) + 0.0488; r² = 0.9802).

Total flavonoid content was determined via an aluminum chloride colorimetric method.^37^ The supernatant was combined with AlCl₃, potassium acetate, and distilled water. After 40 minutes, absorbance was read at 420 nm. A quercetin standard curve was used (Absorbance = 0.0091 × Quercetin (µg) + 0.0206; r² = 0.995).

DPPH radical scavenging capacity was evaluated.^37^ A 50 µL sample of the supernatant was added to 1 mL of a 0.004% (w/v) methanolic DPPH solution. Following a 30-minute dark incubation, absorbance was recorded at 517 nm. The scavenging activity was expressed as a percentage of inhibition: I% = [(A_control−A_sample)/A_control] × 100.

### 2.6. Data analysis

Results are shown as the mean ± standard error (SE) from three replicates. Data were analyzed with Minitab 16.0 software. Treatment means were compared using Tukey's test at a 5% probability level (P ≤ 0.05). Charts were prepared using Microsoft Office Excel 2013.

## 3. Results

### 3.1. Shoot biomass and elongation

**Figure 1.**
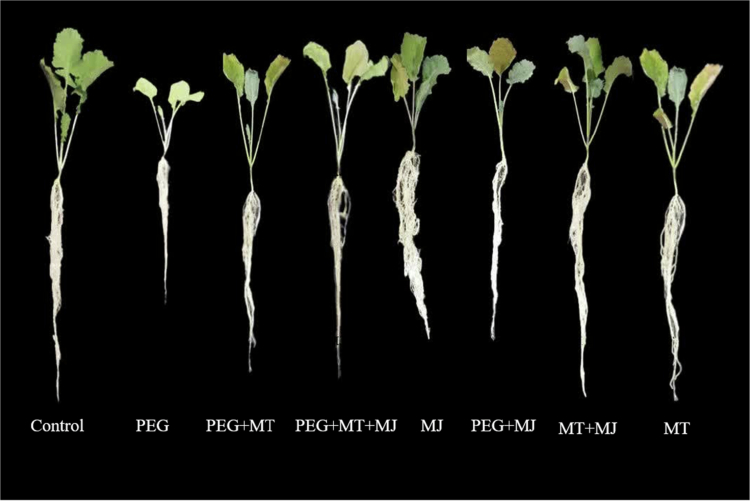
Brassica napus plants subjected to the various experimental treatments: Control, Drought (PEG), Drought + Melatonin (MT), Drought + Methyl Jasmonate (MJ), and Drought + MT + MJ.

**Figure 2.**
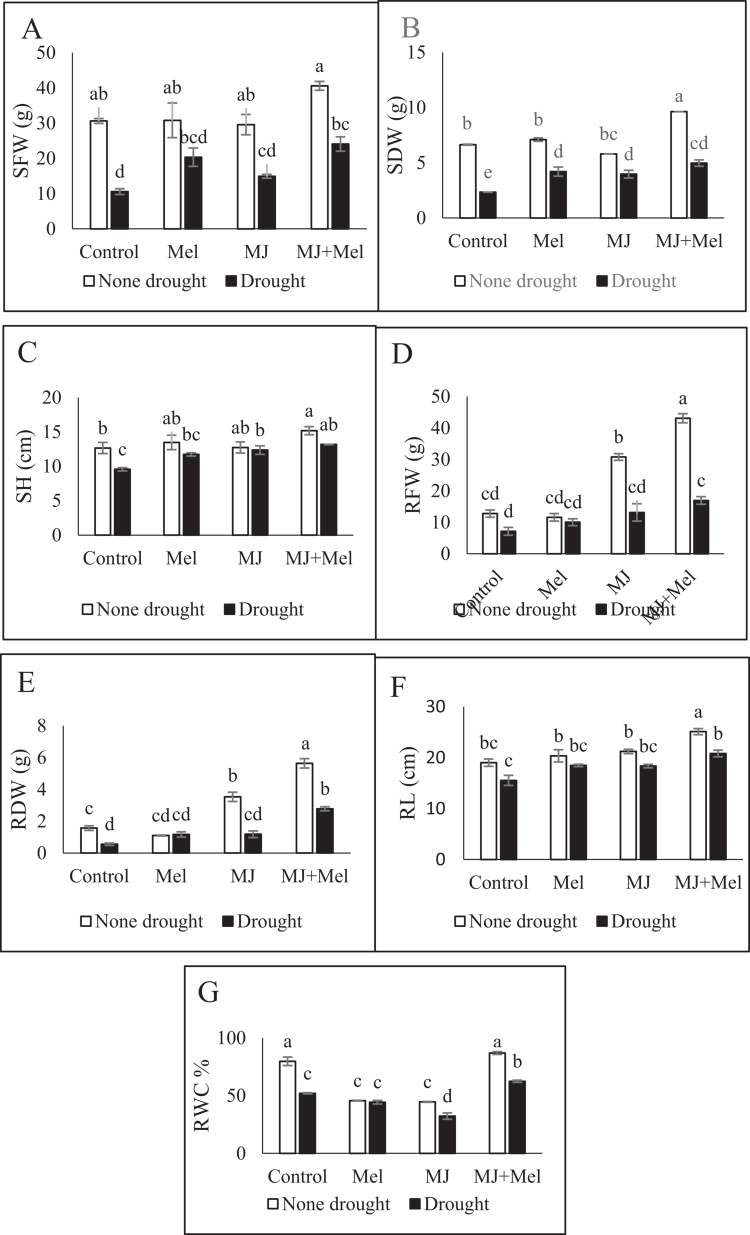
Changes in the SFW (A), SDW (B), SL (C), RFW (D), RDW (E), RL (F) and RWC (G) of rapeseed (Brassica napus) under drought stress conditions and treatments with MT and MJ. Data are presented as the mean ± standard error (SE) in triplicate. The same letters indicate no significant difference at the *p* < 0.05 level. MT: Melatonin, MJ: Methyl jasmonate; MT + MJ: Melatonin + Methyl jasmonate; SFW: shoot fresh weight; SDW: Shoot dry weight; SH: Shoot length; RFW: Root fresh weight; RDW: Root dry weight; RL: Root length; RWC: Relative water content.

Exposure to drought conditions resulted in significant declines in shoot fresh weight (65.49%), dry weight (64.83%), and length (13.18%) relative to well-watered plants. The application of MT enhanced shoot fresh weight, whereas MJ reduced it compared to the unstressed control. Under drought stress, however, both treatments mitigated the negative effects. MT application increased shoot fresh weight, dry weight, and length by 92.33%, 80.25%, and 22.66%, respectively, compared to stressed plants receiving no regulators. MJ application led to increases of 41.15%, 69.95%, and 29.28% in the same parameters. The most pronounced improvement was observed with the combined application of both regulators, yielding the highest recorded values for shoot fresh weight (40.57 g), dry weight (4.96 g), and length (15.17 cm) (Figures 1 and 2A, B, C).

### 3.2. Root biomass and elongation

Drought stress similarly reduced root fresh weight (44.25%), dry weight (64.89%), and length (18.26%). Foliar treatments with MT, MJ, and their combination under drought stress enhanced root growth. Increases in root fresh weight were 28.45%, 66.84%, and 138.11%, respectively. Corresponding increases in root dry weight were 110%, 114%, and 405%. Root length was increased by 18.93%, 18.02%, and 33.99% for each treatment, respectively. The combined MT and MJ treatment demonstrated the most substantial positive impact on all measured root growth parameters (Figure 2 D, E, F).

### 3.3. Relative water content (RWC)

The results indicated that MT and MJ treatments, both under normal and stress conditions, decreased the RWC content. In contrast, this index showed an increase in the MT + MJ treatment compared to the control group (Figure 2G).

### 3.4. Photosynthetic characteristics: pigments, gas exchange, and PSII efficiency

**Figure 3.**
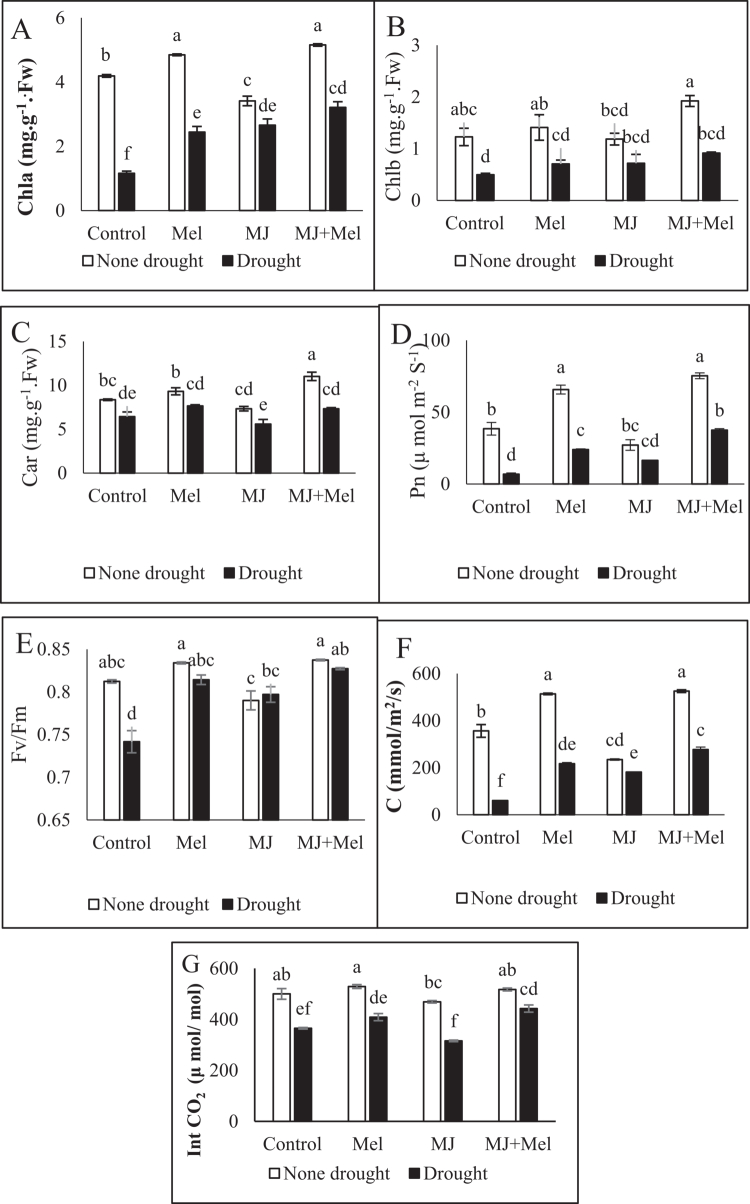
Chl a content (A Chl b (B), Car (C), Pn (D), Fv/Fm (E), C (F) Ci (G) of rapeseed (Brassica napus) under drought stress conditions and treatments with MT and MJ. Data are presented as the mean ± standard error (SE) in triplicate. The same letters indicate no significant difference at the *p* < 0.05 level. MT: Melatonin, MJ: Methyl jasmonate; MT + MJ: Melatonin + Methyl jasmonate; Chl a: Chlorophyll a; Chl b: Chlorophyll b; Car: Carotenoid; Pn: Net photosynthesis; Fv/Fm: Maximum quantum efficiency of photosystem II; C: Stomatal conductance, Ci: Intercellular CO2 concentration.

Drought exposure significantly reduced photosynthetic pigment levels, decreasing chlorophyll a, chlorophyll b, and carotenoid content by 33.72%, 54.59%, and 23.2%, respectively, relative to the control. Under non-stressed conditions, MT and, more markedly, its combination with MJ elevated pigment concentrations, whereas MJ application alone reduced them. Under drought, treatments with MT, MJ, and particularly their combined application increased chlorophyll a and b content. For carotenoids, MT and the combined treatment increased levels, while MJ alone caused a further decline (Figure 3A, B, C).

Net photosynthetic rate (Pn) was significantly affected by drought and growth regulator treatments (*p* < 0.05). Drought stress severely suppressed Pn by 82.22%, but both regulators alleviated this inhibition. MT was more effective than MJ under drought, improving Pn by 71.45%. The lowest Pn value (6.83 µmol m^−2^ s^−1^) was recorded in drought-stressed plants without regulators, while the highest (75.33 µmol m^−2^ s^−1^) was achieved with the combined MT and MJ application (Figure 3D).

The maximum quantum efficiency of photosystem II (Fv/Fm) was significantly lowered by drought. Exogenous application of both regulators, especially MT, enhanced this parameter. The highest Fv/Fm ratio was recorded in plants receiving the combined treatment (Figure 3E).

Stomatal conductance was substantially reduced under drought stress (83.16% decrease). Exogenous MT was more effective (58.72% improvement) than MJ in mitigating this decline. The most effective treatment was the combination of MT and MJ, which increased stomatal conductance by 76.25% compared to the stressed control (Figure 3F).

Drought stress also led to a 26.95% reduction in intercellular CO₂ concentration (Ci). MT application under stress increased Ci, while MJ decreased it. The simultaneous application of both regulators had the greatest positive impact, raising Ci by 17.48% compared to the drought-stressed control group (Figure 3G).

### 3.5. Membrane permeability and lipid peroxidation

**Figure 4.**
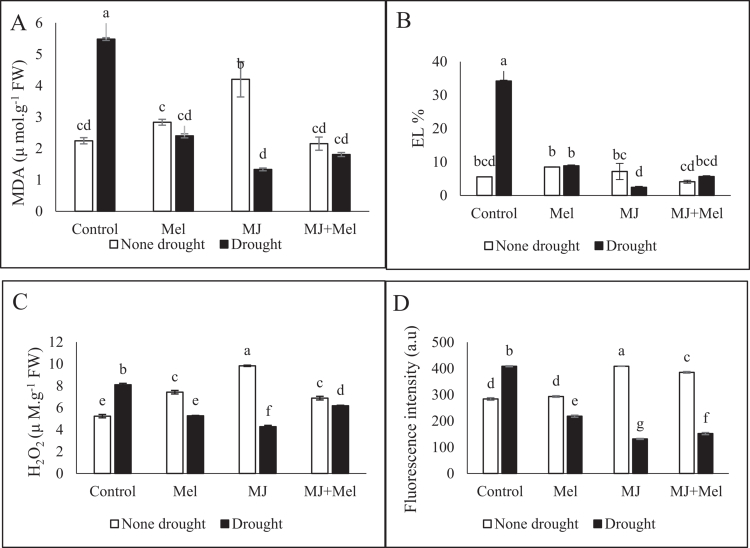
MDA (A), EL (B), H2O2 (C) and ROS (D) changes under drought stress conditions and treatments with MT and MJ. Data are presented as the mean ± standard error (SE) in triplicate. The same letters indicate no significant difference at the *p* < 0.05 level. MT: Melatonin, MJ: Methyl jasmonate; MT + MJ: Melatonin + Methyl jasmonate; MDA: malondialdehyde, EL: Electrolyte leakage; H2O2: Hydrogen peroxide, ROS: reactive oxygen species.

The findings indicated that drought stress induced significant membrane damage, which was reflected in a 514% increase in electrolyte leakage and a 144.6% rise in malondialdehyde (MDA) content compared to the control. The application of growth regulators under stress conditions mitigated this damage, reducing both ion leakage and MDA levels. MJ was notably more effective than MT in this regard. Specifically, MJ application reduced MDA content by 4.12-fold and ion leakage by 13.9-fold. In comparison, MT led to a 2.2-fold decrease in MDA and a 3.8-fold reduction in ion leakage (Figure 4A, B).

### 3.6. Hydrogen peroxide and reactive oxygen species

The data demonstrated that drought stress triggered oxidative stress, elevating the levels of reactive oxygen species (ROS) and hydrogen peroxide (H₂O₂) by 43.96% and 54.79%, respectively. Foliar application of MT and MJ to drought-stressed plants significantly lowered the accumulation of these oxidants. MT treatment reduced ROS and H₂O₂ content by 87.13% and 35.02%, respectively. The reduction was even more pronounced with MJ, which decreased ROS by 210.32% and H₂O₂ by 47.25%. Among the treatments, MJ proved to be the most effective in alleviating oxidative stress under drought conditions (Figure 4C, D).

### 3.7. Compatible osmolytes (proline, total sugar, reducing sugar)

**Figure 5.**
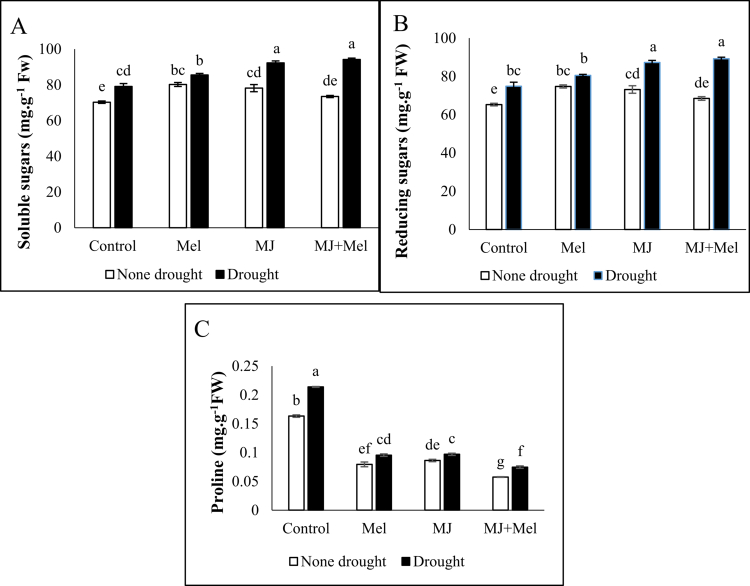
Total sugar (A), Reducing sugar (B) and Proline (C) changes under drought stress conditions and treatments with MT and MJ. Data are presented as the mean ± standard error (SE) in triplicate. The same letters indicate no significant difference at the *p* < 0.05 level. MT: Melatonin, MJ: Methyl jasmonate; MT + MJ: Melatonin + Methyl jasmonate.

Drought stress increased proline content by 23.67% compared to the control group. exogenous application of MT and MJ led to a reduction in proline content. This reduction was 49.10% and 42.89% for MT and MJ, respectively, compared to the control group. The greatest reduction was observed in the group receiving the combined treatment of MT and MJ, where proline content decreased by 86.183% compared to the control group (Figure 5).

### 3.8. Antioxidant metabolites and enzyme activities

**Figure 6.**
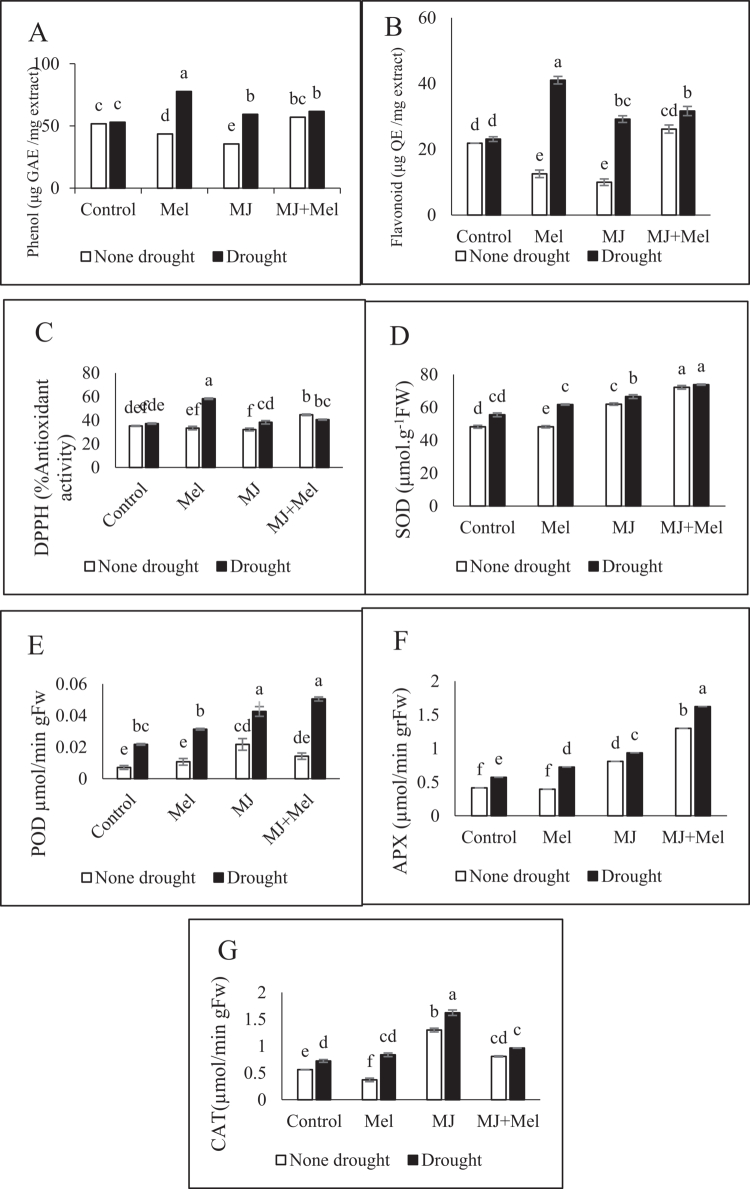
Non-enzymatic (A−C) and Enymatic (D−G) changes under drought stress conditions and treatments with MT and MJ. Data are presented as the mean ± standard error (SE) in triplicate. The same letters indicate no significant difference at the *p* < 0.05 level. MT: Melatonin, MJ: Methyl jasmonate; MT + MJ: Melatonin + Methyl jasmonate; DPPH: 2, 2-Diphenyl-1-picrylhydrazyl, SOD: Superoxide dismutase; POD: Guaiacol Peroxidase; APX: Ascorbate peroxidase; CAT: Catalase.

Under well-watered (non-stressed) conditions, exogenous application of either MT or MJ individually led to a reduction in the concentrations of both phenolic compounds (decreases of 15.91% and 31.43%, respectively) and flavonoids (decreases of 42.65% and 54.32%, respectively). In contrast, the co-application of MT and MJ resulted in elevated levels of these non-enzymatic antioxidants, increasing phenol and flavonoid content by 10.16% and 19.67%, respectively (Figure 6).

Under drought stress, all hormonal treatments enhanced the accumulation of phenolic compounds and flavonoids. The highest levels of total phenolics (77.76 µg gallic acid equivalent per mg extract) and flavonoids (41.29 µg quercetin equivalent per mg extract) were recorded in plants treated with MT (Figure 6).

Drought stress significantly induced the activity of the measured antioxidant enzymes—superoxide dismutase (SOD), peroxidase (POD), ascorbate peroxidase (APX), and catalase (CAT). The most pronounced induction was observed for POD activity, which increased by 216% relative to the unstressed control (Figure 6).

All hormonal treatments applied under drought stress further amplified the activity of these antioxidant enzymes. The combined MT and MJ treatment exerted the strongest enhancing effect on the activities of SOD, POD, and APX. For CAT activity, the highest level was observed with MJ treatment alone (Figure 6).

### 3.9. Heat map correlation (HMC)

**Figure 7.**
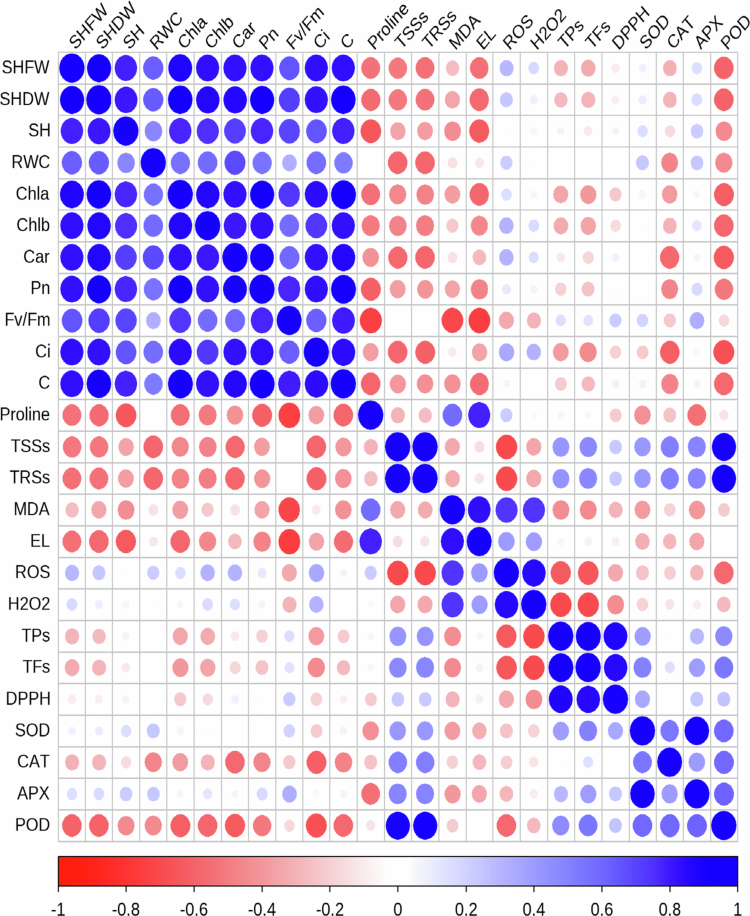
Heatmap illustrating pairwise interactions among measured parameters in Brassica napus based on Pearson’s correlation analysis. Abbreviations: C: Stomatal conductance; Ci: Intercellular CO₂ concentration; TPs: Total phenolics; Car: Carotenoid; Chl: Chlorophyll; FW: Fresh weight; DW: Dry weight; SH: Shoot height; RWC: Relative water content; Pn: Net photosynthesis; Fv/Fm: Maximum quantum efficiency of photosystem II; DPPH: 2, 2-Diphenyl-1-picrylhydrazyl radical scavenging activity; SOD: Superoxide dismutase; CAT: Catalase; APX: Ascorbate peroxidase; TFs: Total flavonoids; POD Guaiacol Peroxidase; TSSs: Total soluble sugars; MDA: Malondialdehyde; TRSs: Total reducing sugars; ROS: Reactive oxygen species; H₂O₂: Hydrogen peroxide; EL: Electrolyte leakage.

The results of the Pearson correlation analysis, visualized via a heatmap, indicated significant positive associations among photosynthetic parameters, growth indices, and both enzymatic and non-enzymatic antioxidants. Inversely, negative correlations were found between these beneficial traits and the concentrations of oxidants and membrane damage indicators (Figure 7).

## 4. Discussion

The present study provides a comprehensive physiological and biochemical evaluation of how exogenous MT and MJ mitigate the detrimental effects of drought stress in *Brassica napus*. Our findings unequivocally demonstrate that while both compounds are effective, their individual and combined applications elicit distinct and often synergistic responses, ultimately leading to enhanced drought tolerance through a coordinated improvement in growth, photosynthetic performance, osmotic adjustment, and oxidative defense.

The severe inhibition of shoot and root growth under drought stress, evidenced by the marked reductions in fresh weight, dry weight, and length, is a classic symptom of water deficit, primarily resulting from impaired cell division and elongation, as well as reduced photosynthetic output.^6^ The remarkable restoration of growth parameters by MT and MeJA, with the combined treatment proving most efficacious, indicates a potent role for these regulators in sustaining metabolic processes under stress. The spectacular increases in root dry weight (405%) and fresh weight (138.11%) with the MT + MeJA treatment are particularly significant. A robust root system is critical for water foraging under drought, and this synergistic enhancement suggests that the two compounds together powerfully stimulate carbon partitioning and root development, a key adaptive strategy for stress avoidance.^38^

This growth promotion is inextricably linked to the preservation of photosynthetic capacity. Drought stress severely disrupted the photosynthetic apparatus, as shown by the declines in chlorophyll content, net photosynthesis (Pn), stomatal conductance (C), and the maximum quantum efficiency of PSII (Fv/Fm). The reduction in intercellular CO₂ concentration (Ci) alongside decreased stomatal conductance suggests that the initial limitation to photosynthesis was primarily stomatal, preventing CO₂ diffusion. However, the sustained low Pn even when some treatments increased C and Ci implies that non-stomatal limitations (e.g., photoinhibition, Rubisco activity) also became significant.^39^ The application of MT and MeJA, especially in combination, effectively counteracted these limitations. MT's superior performance over MeJA in single applications for Pn and Fv/Fm aligns with its known role in protecting chlorophyll from degradation and stabilizing the D1 protein of PSII.^40^ The combined treatment's ability to produce the highest values for all photosynthetic parameters underscores a synergistic interaction, likely by concurrently optimizing stomatal aperture and protecting the photochemical and biochemical phases of photosynthesis.

The primary driver of drought-induced damage is the accumulation of reactive oxygen species (ROS), which we observed as significant increases in H₂O₂ and overall ROS content, leading to oxidative damage manifested as elevated MDA and ion leakage. The differential efficacy of MT and MeJA in mitigating this oxidative burst is a cornerstone of our findings. MeJA was strikingly more effective than MT in scavenging ROS and H₂O₂ and, consequently, in reducing membrane damage (MDA and ion leakage). This potent direct antioxidative effect of MeJA is likely mediated through its profound upregulation of the enzymatic antioxidant system, particularly catalase (CAT), which is highly efficient in detoxifying high levels of H₂O₂.^41^ In contrast, while MT also enhanced antioxidant enzymes, its most prominent role appeared to be in boosting the non-enzymatic antioxidant system, as it led to the highest accumulation of phenols and flavonoids under drought. MT is a known master regulator of the phenylpropanoid pathway, thereby enriching the antioxidant pool.^42^ This functional specialization—where MeJA excels in enzymatic ROS scavenging and MT in non-enzymatic antioxidant biosynthesis—explains the powerful synergy of their combined application, which provided the most comprehensive oxidative defense.

An intriguing and seemingly paradoxical result was the effect on compatible osmolytes. While drought stress typically triggers proline accumulation as an osmotic adjuster, the hormonal treatments, particularly the combination, dramatically reduced proline content. This suggests that the primary mode of action of MT and MeJA is not to enhance osmotic adjustment via proline but to so effectively alleviate the stress burden that the plant no longer needs to invest energy in massive proline synthesis. The improved water status, as indirectly reflected in the RWC trend for the combined treatment and the direct evidence from reduced oxidative stress and improved photosynthesis, means the osmotic crisis is averted. The reduction in proline could therefore be interpreted as a marker of successful stress mitigation, allowing the plant to revert to a more growth-oriented metabolic state.^43^

The condition-dependent effects on secondary metabolites under non-stress conditions further highlight the complex signaling interplay. The suppression of phenols and flavonoids by individual MT or MeJA treatments suggests a reallocation of resources away from defense and towards growth in the absence of stress. However, the synergistic induction by their combination indicates a unique priming effect, where the concurrent signaling pre-arms the plant's chemical defenses, a phenomenon that likely contributed to its superior performance under subsequent drought stress.^44^

**Figure 8.**
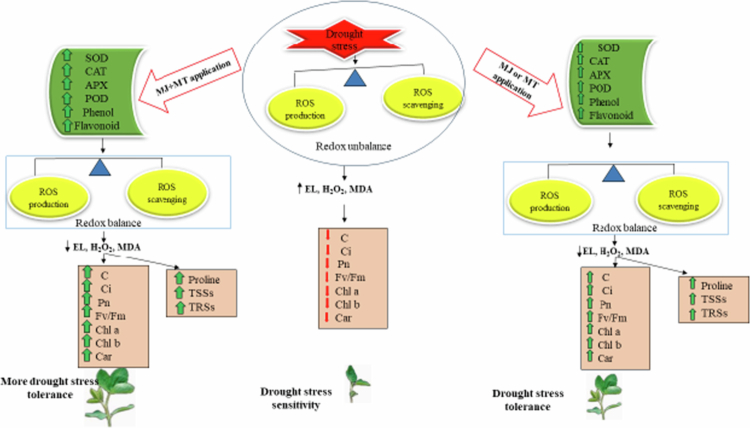
The diagram depicts the negative impact of drought on the redox equilibrium of *Brassica napus*, mediated by changes in morphological, physiological, and photosynthetic traits. Conversely, it demonstrates the protective, tolerance-inducing roles of individual and combined applications of MT and MJ. The arrow thickness corresponds to the magnitude of change in the measured parameters.

Finally, the Pearson correlation heatmap provides a holistic validation of these interconnected physiological responses. The strong positive correlations among growth, photosynthetic parameters, and antioxidants, and their negative correlations with oxidative markers, elegantly synthesize our central thesis: the application of MT and MeJA, particularly in concert, establishes a positive feedback loop. By robustly enhancing the antioxidant system (both enzymatic and non-enzymatic), these regulators minimize oxidative damage, which in turn preserves photosynthetic machinery and membrane integrity, thereby sustaining growth and development under drought stress conditions (see Figure 8).

In conclusion, our results demonstrate that MT and MJ act as potent mitigators of drought stress through complementary and synergistic mechanisms. MT primarily excels in preserving photosynthetic function and boosting non-enzymatic antioxidants, whereas MJ is a more potent inducer of the enzymatic antioxidant system, leading to superior direct ROS scavenging. Their combined application harnesses these strengths, resulting in a more resilient phenotype characterized by enhanced root and shoot growth, superior photosynthetic capacity, and a profoundly enhanced capacity to manage oxidative stress, ultimately conferring a greater level of drought tolerance in *Brassica napus*. Our findings unequivocally demonstrate that while both compounds are effective, their individual and combined applications elicit distinct and often synergistic responses, ultimately leading to enhanced drought tolerance through a coordinated improvement in growth, photosynthetic performance, osmotic adjustment, and oxidative defense.

The findings of this study hold significant promise for agricultural practice and future research. From a practical standpoint, the synergistic foliar application of melatonin (MT) and methyl jasmonate (MJ) offers a viable, eco-friendly strategy to enhance drought resilience in rapeseed (Brassica napus). This approach can be directly adopted by farmers in drought-prone regions, such as Iran, to mitigate yield losses without heavy reliance on increased irrigation. By preserving photosynthetic function and bolstering antioxidant defenses, this treatment helps maintain both crop biomass and seed quality under water deficit. It represents a cost-effective biostimulant regimen that aligns with sustainable agriculture goals, potentially reducing water and chemical inputs while stabilizing oilseed production in the face of climate variability.

Looking forward, several avenues for research emerge. First, field-scale trials are essential to validate these greenhouse results under real-world agronomic conditions, assessing performance across different soil types, climates, and rapeseed cultivars. Second, deeper mechanistic investigation into the molecular crosstalk between MT and MJ signaling pathways is warranted, potentially using transcriptomic or proteomic approaches to identify key regulatory genes and proteins. Third, research should explore the optimal timing, frequency, and formulation of application—including seed priming or nanoparticle delivery—to maximize efficacy and farmer adoption. Fourth, the combined treatment’s potential should be tested against other abiotic stresses (e.g., salinity, heat) and in other economically important crops to evaluate its broader applicability. Finally, long-term studies on soil health, environmental impact, and economic feasibility will be crucial for integrating this strategy into sustainable cropping systems. Together, these directions can translate a promising physiological intervention into a practical, scalable solution for climate-resilient agriculture.

## Data Availability

Data is provided within the manuscript. All datasets generated or analyzed during this study are available from the corresponding author upon reasonable request.
